# Alcohol consumption, smoking and overweight as a burden for health care services utilization: a cross-sectional study in Estonia

**DOI:** 10.1186/1471-2458-13-772

**Published:** 2013-08-23

**Authors:** Kaire Vals, Raul-Allan Kiivet, Mall Leinsalu

**Affiliations:** 1 Department of Public Health, University of Tartu, Ravila 19, Tartu 50411, Estonia; 2 Infectious Diseases and Drug Monitoring Department, National Institute for Health Development, Tallinn, Estonia; 3 Stockholm Centre on Health of Societies in Transition, Södertörn University, Stockholm, Sweden; 4 Department of Epidemiology and Biostatistics, National Institute for Health Development, Tallinn, Estonia

**Keywords:** Health care utilization, Smoking, Alcohol, Overweight, Obesity

## Abstract

**Background:**

Alcohol consumption, smoking and weight problems are common risk factors for different health problems. We examine how these risk factors are associated with the use of health care services.

**Methods:**

Data for 6500 individuals in the 25–64 age group came from three cross-sectional postal surveys conducted in 2004, 2006, and 2008 in Estonia. The effect of alcohol consumption, smoking and weight problems on the use of primary and specialist care services, hospitalizations and ambulance calls was analysed separately for men and women by using binary logistic regression.

**Results:**

Overweight and/or obesity were strongly related to the use of primary care and out-patient specialist services for both genders, and to hospitalizations and ambulance calls for women. Current smoking was related to ambulance calls for both genders, whereas smoking in the past was related to the use of primary care and specialist services among men and to hospitalizations among women. Beer drinking was negatively associated with all types of health care services and similar association was found between wine drinking and hospitalizations. Wine drinking was positively related to specialist visits. The frequent drinking of strong alcohol led to an increased risk for ambulance calls. Drinking light alcoholic drinks was positively associated with all types of health care services (except ambulance calls) among men and with the use of specialist services among women.

**Conclusions:**

Overweight and smoking had the largest impact on health care utilization in Estonia. Considering the high prevalence of these behavioural risk factors, health policies should prioritize preventive programs that promote healthy lifestyles in order to decrease the disease burden and to reduce health care costs.

## Background

Alcohol consumption, smoking and weight problems are common risk factors for different non-communicable diseases such as cardiovascular diseases, type 2 diabetes, pulmonary and orthopaedic diseases and cancers, and also for injuries [1-4]. These risk factors, through their links with consequent health problems incur a large cost for society through premature mortality and extended costs on medical care [2,5,6]. Sturm, for example has estimated that as much as 36% increase in in- and outpatient spending and 77% increase in medications can be attributed to weight problems, and 21% and 28% respectively to smoking [5]. Haapanen-Niemi found that male smokers had up to 70% more hospital days due to health conditions compared to those who had never smoked [7]. Problem drinking has mainly been related to the extended use of acute medical services [8,9].

In Estonia about 33% of the total disease burden is attributable to cardiovascular diseases, 20% to cancers, and 12% to external causes of death such as traffic accidents, falls, suicides and homicides [10], all at least partly affected by unhealthy behaviours. Smoking, alcohol consumption, overweight and obesity are highly prevalent among the general population in Estonia [11]. Since the 1990s, an increase has been reported for alcohol consumption and obesity (the latter only among men) [12,13]. The increasing prevalence of obesity follows similar trends in other countries [14,15]. At the same time the prevalence of daily smoking has decreased in recent years [11], with a steeper decline observed in men as predicted by the progression of the tobacco epidemic [16].

The Estonian health care system is funded on a solidarity basis. Medical expenditures incurred by children, retired persons and employees are paid by the Estonian Health Insurance Fund (EHIF) which is financed through a social security tax (34.4% of gross earnings). About 95% of the Estonian population is covered by the EHIF [17]. In accordance with overall economic growth, the total per capita costs of the health sector doubled between 2004 and 2008. The share of health expenditures as a percentage of GDP increased from 5.2% in 2004 to 6.1% in 2008 with up to a 10% increase occurring in the number of contacts with health care services over this period [18]. Previous studies in Estonia have demonstrated a strong effect of socioeconomic factors on the use of health care services [19,20] with more deprived groups being less likely to use primary and specialist care or to visit the dentist when compared to those with higher social status. These studies, however, did not look at the impact of unhealthy behaviours that could underlie the observed differences. Such behaviours may have an independent effect on health care utilization, as shown for obesity in a recent study by Tekkel et al [13], and in this way, impose a heavy burden on health care in society.

The aim of this study is to analyse the association between alcohol consumption, smoking and weight problems with the use of health care services among the adult population in Estonia. This knowledge will improve our understanding of the underlying causes of health care utilization and thus help the health authorities to better ground their strategies, which is essential for the more effective use of health care resources.

## Methods

### Data

The data were drawn from the Health Behaviour Survey of the Estonian Adult Population, a nationally representative cross-sectional postal survey. The survey is part of the collaborative Finbalt Health Monitoring project described in detail elsewhere [21]. In Estonia, the survey has been conducted in every second year since 1990 and the data are freely available upon request. A simple random sample of the Estonian population aged 16–64 years has been drawn from the population register for each survey. The questionnaires were compiled in both Estonian and Russian and were for the most part mailed in April and May. After two weeks a first reminder was sent to those who had not responded, and a second reminder was sent after another two weeks. We combined data from three surveys conducted in 2004, 2006 and 2008 in order to increase statistical power. The adjusted response rate was 62% in 2004 (n = 3074), 57% in 2006 (n = 2867) and 62% in 2008 (n = 3004) [11,22,23]. We excluded respondents younger than 25 years of age as this group might not yet have achieved their highest educational level which could result in their socioeconomic status being underestimated compared to older respondents. All respondents who had missing values for any of the study variables were additionally excluded from the final sample which consisted of 6500 individuals in the 25–64 age group.

### Outcome variables

Utilization of health care was studied for four types of services. The first indicator measures the proportion of respondents who had visited a general practitioner (GP) over the past 12 months. This variable covers visits at the primary care level. The second variable covers visits to out-patient specialists over the past 12 months, excluding dentists. The third indicator measures the proportion of respondents who had been hospitalized (for any reason) during the 12 months preceding the survey. The fourth indicator measures the proportion of respondents who had made an ambulance call over the past 12 months.

### Independent variables

The association with health care utilization was assessed for alcohol consumption, smoking and weight problems. Alcohol consumption was measured by the average frequency of drinking in the past 12 months separately for strong alcohol, wine, beer, and light alcoholic drinks. The answer categories were divided into three groups for each type of beverage: has not drunk (reference group), has drunk a few times a month or less (combining the categories ‘a few times a year’ and ‘a few times a month’), and has drunk at least a few times a week (combining the categories ‘a few times a week’ and ‘every day’). For smoking the answer categories were combined into three groups: those who had never smoked (reference group), those who had smoked in the past, and current smokers. Occasional smokers were classified as current smokers as we were not able to classify them as never smokers or ex-smokers. Weight problems were assessed on the basis of body mass index calculated from self-reported height and weight [BMI = weight (kg)/height (m)^2^]. Respondents were divided into four categories: underweight (BMI < 18.50), normal weight (18.50–24.99), overweight (25.00–29.99), and obese (≥30.00). The normal weight category was used as a reference group.

### Control variables

In order to control for the possible effect of confounding by socioeconomic variables, we adjusted our analysis for education (categorized as university, upper secondary and lower secondary education), average household income (divided into high, mid and low income groups) and self-defined ethnic identity (Estonian, Russian and other). These indicators are known from previous studies to be related to both health care use and to health behaviours [12,13,20,24]. We additionally adjusted models for age and for study year, the latter to control for any period effect.

### Statistical analysis

The data were analysed separately for men and women. Relative differences were evaluated by means of binary logistic regression using the STATA 12 statistical package. The effect of each independent variable was measured in two models. The first model included only age (in 5-year age groups) and the risk factor of interest. The second model was mutually adjusted for age, alcohol consumption (all four measures), smoking and BMI, as well as for education, income, ethnicity and study year. The differences between the two models were small and we therefore show the results only for the multivariate models. The results are presented as odds ratios (ORs) with 95% confidence intervals (CIs).

### Ethics committee approval

The surveys have been approved by the Tallinn Medical Research Ethics Committee. The data used in the current study are anonymous.

## Results

The sample characteristics are presented in Table 1. Alcohol consumption, smoking and weight problems were all highly prevalent among respondents. Nearly 16% of men drank strong alcohol and 42% drank beer at least a few times a week. Almost 8% of women drank wine with the same frequency. 78% of men and 48% of women had been smokers at some period in their life and 59% of men and 48% of women were overweight or obese. Large gender differences were present for alcohol consumption and smoking, whereas these differences were modest for obesity. Women tended to use primary health care and out-patient specialist services more often than men.

**Table 1:** Characteristics of the study population

	**Men (N = 2699)**		**Women (N = 3801)**	
	**N**	**%**	**N**	**%**
Strong alcohol consumption in past12 months				
Has not drunk	399	14.8	1345	35.4
A few times a month or less	1878	69.6	2357	62.0
At least a few times a week	422	15.6	99	2.6
Wine consumption in past 12 months				
Has not drunk	1223	45.3	988	26.0
A few times a month or less	1281	47.5	2520	66.3
At least a few times a week	195	7.2	293	7.7
Beer consumption in past 12 months				
Has not drunk	640	23.7	2214	58.3
A few times a month or less	930	34.5	1313	34.5
At least a few times a week	1129	41.8	274	7.2
Light alcoholic drinks consumption in past 12 months				
Has not drunk	1780	66.0	2092	55.0
A few times a month or less	763	28.2	1528	40.2
At least a few times a week	156	5.8	181	4.8
Smoking				
Has never smoked	607	22.5	1976	52.0
Has smoked in the past	771	28.6	791	20.8
Current smoker	1321	48.9	1034	27.2
BMI				
Normal weight (18.5–24.99)	1090	40.4	1863	49.0
Underweight (<18.50)	19	0.7	100	2.6
Overweight (25–29.99)	1103	40.9	1114	29.3
Obesity (≥30)	487	18.0	724	19.1
General practitioner				
No	941	34.9	973	25.6
Yes	1758	65.1	2828	74.4
Specialized care				
No	1754	65.0	1702	44.8
Yes	945	35.0	2099	55.2
Hospitalization				
No	2416	89.5	3387	89.1
Yes	283	10.5	414	10.9
Ambulance calls				
No	2517	93.3	3551	93.4
Yes	182	6.7	250	6.6
Education				
University	559	20.7	1091	28.7
Upper secondary	1699	63.0	2347	61.7
Lower secondary	441	16.3	363	9.6
Household income				
Higher 1/3	873	32.4	977	25.7
Mid 1/3	1005	37.2	1515	40.0
Lower 1/3	821	30.4	1309	34.3
Ethnicity				
Estonians	1906	70.6	2619	68.9
Russians	644	23.9	960	25.3
Other	149	5.5	222	5.8
Age group				
25-29	333	12.3	422	11.1
30-34	355	13.2	485	12.8
35-39	312	11.6	450	11.8
40-44	354	13.1	455	12.0
45-49	338	12.5	514	13.5
50-54	360	13.3	508	13.4
55-59	314	11.6	495	13.0
60-64	333	12.4	472	12.4

Table 2 presents the results for the use of primary care and out-patient specialist services. The consumption of strong alcohol or wine was not related to the use of primary care services in either men and women. Drinking beer or light alcoholic drinks was not related to the use of primary care services among women, however, men who drank beer at least a few times a week were less likely to visit a general practitioner (GP), and men who drank light alcoholic drinks a few times a week were more likely (OR = 1.57) to visit a GP compared to those who did not drink these beverages. Smoking in the past was related to the use of primary care services among men but not women. Both overweight and obesity were positively related to the use of primary care services in men and women. The odds of visiting a GP were almost 80% higher for obese women and 32% higher for obese men than for those who were a normal weight.

**Table 2:** The association^* ^**of alcohol consumption, smoking and weight problems with the use of primary care and out-patient specialist services**

	**Use of primary care services**		**Use of out-patient specialist services**	
	**Men**	**Women**	**Men**	**Women**
	**OR (95% CI)**	**OR (95% CI)**	**OR (95% CI)**	**OR (95% CI)**
Strong alcohol consumption in past 12 months				
Has not drunk	1	1	1	1
A few times a month or less	1.02 (0.80-1.32)	1.13 (0.96-1.33)	1.17 (0.91-1.51)	1.09 (0.94-1.26)
At least a few times a week	0.85 (0.63-1.16)	0.71 (0.45-1.12)	1.34 (0.98-1.83)	1.11 (0.73-1.72)
Wine consumption in past 12 months				
Has not drunk	1	1	1	1
A few times a month or less	0.97 (0.80-1.18)	0.93 (0.77-1.12)	1.21 (1.00-1.48)	1.24 (1.05-1.46)
At least a few times a week	1.02 (0.72-1.43)	1.04 (0.75-1.44)	1.23 (0.87-1.72)	1.05 (0.79-1.41)
Beer consumption in past 12 months				
Has not drunk	1	1	1	1
A few times a month or less	0.88 (0.69-1.12)	0.86 (0.73-1.02)	0.86 (0.68-1.09)	0.96 (0.82-1.11)
At least a few times a week	0.71 (0.56-0.90)	0.83 (0.62-1.12)	0.65 (0.52-0.82)	0.76 (0.58-0.99)
Light alcoholic drinks consumption in past 12 months				
Has not drunk	1	1	1	1
A few times a month or less	1.21 (0.98-1.48)	1.01 (0.86-1.20)	1.23 (1.00-1.51)	1.24 (1.07-1.44)
At least a few times a week	1.57 (1.08-2.29)	1.28 (0.88-1.86)	1.12 (0.78-1.60)	1.11 (0.80-1.53)
Smoking				
Has never smoked	1	1	1	1
Has smoked in the past	1.43 (1.13-1.81)	0.96 (0.79-1.16)	1.26 (1.01-1.58)	1.03 (0.87-1.23)
Current smoker	1.12 (0.90-1.39)	0.99 (0.82-1.19)	0.81 (0.65-1.01)	0.84 (0.71-0.99)
BMI				
Normal weight (18.5–24.99)	1	1	1	1
Underweight (<18.50)	1.27 (0.47-3.45)	1.06 (0.67-1.68)	1.52 (0.59-3.92)	1.20 (0.78-1.85)
Overweight (25–29.99)	1.21 (1.01-1.45)	1.19 (1.00-1.43)	1.09 (0.91-1.31)	1.00 (0.85-1.17)
Obesity (≥30)	1.32 (1.04-1.68)	1.76 (1.40-2.21)	1.28 (1.01-1.61)	1.28 (1.06-1.55)

^*^ Models are mutually adjusted for age, alcohol consumption (all four variables), smoking, BMI, education, income, ethnicity and study year.

Drinking strong alcohol was also not related to the use of outpatient specialist care services on a statistically significant level, though a somewhat (OR = 1.34) elevated risk was observed for men who drank strong alcohol at least a few times a week. Drinking wine or light alcoholic drinks a few times a month or less was associated with a 21-24% increase in the odds of visiting a specialist for both genders, whereas beer drinking was negatively associated with specialist care use. While male ex-smokers used specialist care more often (OR = 1.26) than never smokers, current smoking had a negative association (among women on a statistically significant level) with specialist care use. Both obese men and women had 28% higher odds of visiting a specialist compared to respondents whose weight was normal.

Table 3 combines the results for hospitalizations and ambulance calls. Except for drinking light alcoholic drinks at least a few times a week (OR = 1.81, men only), alcohol consumption was not related with a higher risk for hospitalizations. In contrast, wine drinking was negatively associated with hospitalizations among both men and women. Women, who smoked in the past had nearly 50% higher odds for hospitalizations compared to never smokers, but no association was observed with current smoking for either genders. Obese women had more than 50% higher odds for hospitalizations, but no association was observed among men who were either overweight or obese.

**Table 3:** The association^* ^**of alcohol consumption, smoking and weight problems with hospitalizations and ambulance calls**

	**Hospitalizations**		**Ambulance calls**	
	**Men**	**Women**	**Men**	**Women**
	**OR (95a% CI)**	**OR (95% CI)**	**OR (95% CI)**	**OR (95% CI)**
Strong alcohol consumption in past 12 months				
Has not drunk	1	1	1	1
A few times a month or less	0.84 (0.58-1.20)	0.81 (0.65-1.02)	1.06 (0.68-1.68)	1.00 (0.75-1.33)
At least a few times a week	1.19 (0.76-1.84)	0.86 (0.43-1.74)	1.35 (0.79-2.31)	2.10 (1.05-4.22)
Wine consumption in past 12 months				
Has not drunk	1	1	1	1
A few times a month or less	0.87 (0.64-1.17)	0.74 (0.58-0.94)	0.70 (0.48-1.02)	0.88 (0.65-1.20)
At least a few times a week	0.42 (0.21-0.84)	0.54 (0.32-0.92)	0.74 (0.37-1.51)	0.61 (0.31-1.19)
Beer consumption in past 12 months				
Has not drunk	1	1	1	1
A few times a month or less	1.05 (0.74-1.49)	0.94 (0.73-1.19)	0.88 (0.57-1.34)	0.95 (0.70-1.29)
At least a few times a week	0.76 (0.53-1.07)	0.79 (0.50-1.27)	0.66 (0.43-1.00)	0.87 (0.50-1.52)
Light alcoholic drinks consumption in past 12 months				
Has not drunk	1	1	1	1
A few times a month or less	1.21 (0.87-1.70)	0.87 (0.68-1.10)	1.27 (0.84-1.92)	1.06 (0.78-1.43)
At least a few times a week	1.81 (1.08-3.03)	0.90 (0.51-1.57)	1.05 (0.51-2.17)	1.41 (0.76-2.61)
Smoking				
Has never smoked	1	1	1	1
Has smoked in the past	0.94 (0.65-1.35)	1.47 (1.13-1.90)	1.16 (0.70-1.94)	1.38 (0.98-1.94)
Current smoker	0.95 (0.68-1.34)	1.05 (0.80-1.36)	1.63 (1.03-2.57)	1.38 (1.00-1.90)
BMI				
Normal weight (18.5–24.99)	1	1	1	1
Underweight (<18.50)	1.88 (0.59-5.96)	1.54 (0.85-2.81)	1.96 (0.54-7.17)	2.51 (1.30-4.88)
Overweight (25–29.99)	0.75 (0.56-1.00)	1.17 (0.91-1.52)	0.86 (0.61-1.22)	1.13 (0.81-1.58)
Obesity (≥30)	0.95 (0.67-1.36)	1.54 (1.16-2.05)	0.79 (0.50-1.26)	1.45 (1.01-2.07)

^*^ Models are mutually adjusted for age, alcohol consumption (all four variables), smoking, BMI, education, income, ethnicity and study year.

Drinking strong alcohol at least a few times a week was associated with a two times (OR = 2.10) higher risk for ambulance calls among women compared to those women who had not drunk strong alcohol. Though slightly elevated (OR = 1.35), this association was not statistically significant for men. Frequent consumption of wine or beer showed a negative association (statistically not significant) with ambulance calls. Both men and women who were current smokers had higher odds for ambulance calls compared to never smokers (respective odds ratios 1.63 and 1.38). Women who had smoked in the past also had an elevated risk (OR = 1.38) but the association was not statistically significant. Both obesity and underweight (OR = 1.45; OR = 2.51) increased the risk for ambulance calls among women, whereas a much higher risk in men who were underweight was not statistically significant.

## Discussion

Our study showed that alcohol consumption, smoking and weight problems were associated with the health care utilization, though the results varied by gender and type of services. Overweight and/or obesity were strongly related with the use of primary care and out-patient specialist services for both genders, and with hospitalizations and ambulance calls for women. Current smoking was strongly related to ambulance calls for both genders, whereas smoking in the past was related to the use of primary care and specialist services among men and to hospitalizations among women. Beer drinking was negatively associated with all types of health care services use and a similar association was found between wine drinking and hospitalizations. At the same time wine drinking was positively related to specialist visits. The frequent drinking of strong alcohol led to an increased risk for ambulance calls. Drinking light alcoholic drinks was positively associated with all types of health care services (except ambulance calls) among men and with the use of specialist services among women.

This study has several limitations which should be noted before the discussion of the main findings. First, the survey response rate varied between 57% in 2006 and 62% in 2004 and 2008. In Estonia, a relatively large part of the survey non-response is related to an inability to reach respondents at a given address [22,23], which to some extent reflects the poorly recorded out-migration in recent years. Non-response tended to be slightly higher among men and in the younger age groups across all surveys covered in this study. The results are biased only if non-respondents systematically differ from respondents. Respondents usually tend to have better health and healthier lifestyles compared to non-respondents [25] but there is no particular reason to believe that non-respondents who engage in unhealthy behaviours or who are in worse health use health services differently than respondents with the same characteristics, though some underestimation of the associations is possible. Previous research has also shown that non-response is unlikely to affect the results in terms of the studied associations [25]. Second, the general propensity of respondents to give socially desirable answers, may have led to the underreporting of weight (and consequently the BMI), smoking or alcohol consumption [26-28]. In that case, some underestimation of the associations is possible. Similarly, by classifying occasional smokers as current smokers we may have somewhat underestimated the association between current smoking and the use of health care services. Third, we excluded all cases with missing values in our analyses. However, comparing the results from the bivariate analysis (data not shown) revealed no major differences between the samples with or without the exclusion of cases with missing values. In conclusion, although we cannot exclude the possibility of some underestimation of the associations, we believe that our results generally are not biased in any major way.

The detrimental effect of alcohol on numerous causes of death has increasingly been reported, particularly in relation to Eastern Europe [29]. The harmful effects of alcohol, both chronic and acute, are likely to annul any positive effect of alcohol on health [4]. The estimated social costs of alcohol misuse (1.6% of GDP), including the costs to the health care system are high in Estonia compared to many other countries [30]. Previous findings of the effect of alcohol consumption on health care services utilization have not been consistent. Most studies have reported the negative association between alcohol consumption and the use of out-patient health care services [8,31,32]. Similar to these findings, the frequent consumption of alcohol has also been related to better self-rated health [33]. For in-patient services some studies have reported an inverse relationship [32,34] whereas some studies have found increased usage among problem drinkers [8,9,31]. The latter has mostly been related to the extended use of acute medical services [8,9,35] or to certain health problems like injuries and accidents [7]. Life-time abstainers have consistently been found to use health services more often than moderate drinkers [31,36], which suggests that the association may have a U-shaped form. To a large extent our results accord with previous studies i.e. alcohol consumption is largely associated with a diminished use of both in- and out-patient health care services, though frequent consumption of strong alcohol was related to ambulance calls and wine consumption was related to the use of out-patient specialist care. The inverse association between drinking and health care use may be partly related to the fact that persons with the most serious drinking problems are not participating in the surveys. They may also be more careless about their health and less willing to use health care services [7], especially general practitioners. Missed care, however, may lead to more serious and often fatal health outcomes which in turn would lay an even higher economic burden on society. This could also explain the different pattern we found for ambulance calls. In Estonia, like in other countries of the former Soviet Union, alcohol consumption is considered as a normative male behaviour [37]. Non-drinking in such circumstances may be related to poor health status and more frequent use of health care, which may partly explain the inverse associations with health care use in our study. It is also possible that the group of non-drinkers in our study included ex-drinkers who had stopped drinking for health reasons. The strong positive association between the consumption of light alcoholic drinks and the use of in-and out-patient health care services, especially among men, is interesting but needs more research. Not being a typical male drink, light alcohol may be used as a replacement for strong alcohol after the occurrence of health problems or it may be related to different health risks affecting the use of health services.

Smoking is one of the most serious threats for several health conditions [38]. In 2005, 16% of all deaths in adults over 30 years in the WHO European Region were due to tobacco [39]. Tobacco is responsible for 10% of all deaths from cardiovascular diseases, 22% of all cancer deaths, and 36% of all deaths from diseases of the respiratory system [39]. This implies that health care costs for smokers are higher than for non-smokers [2]. It has been shown that the increased medical costs among smokers were mainly attributable to the increased use of inpatient medical care [40]. Several other studies have shown that current smoking or smoking in the past greatly increases the number of hospitalizations and days spent in hospital [7,9,32,41,42]. This is different for out-patient health care where it has been found that current smokers use services less or similarly to never-smokers [40-43]. Jorm additionally reported that current smokers were less likely to use primary care services where out-of-pocket payment was necessary, and less likely to use some preventive services [43]. Former smokers have been found to use out-patient services more than never-smokers [32,41]. In line with these results, current smoking also showed a strong and positive association with ambulance calls for both genders in our study, although hospitalizations were related to former smoking only among women. Considering the high prevalence of smoking and the high burden of smoking related diseases in Estonia [39], it is surprising that neither current or past smoking were related to hospitalizations among men. In contrast to many other studies [7,40], our study used a cross-sectional design which implies that only survivors were represented among past hospital users. Higher lethality among smokers compared to never-smokers could possibly explain why we did not find any differences in hospitalizations between smokers, ex-smokers and never smokers. On the other hand, the occurrence of health problems may have led to smokers quitting [44], thus explaining the positive association with primary and specialized care use by former male smokers. Current smoking was not related to the use of primary care, although women who were current smokers used specialist care less often than never-smokers. Similar to alcohol drinking, tobacco smoking has been related to enjoyment and stress relief [45] which may explain why current smoking is not always associated with poor subjective perceptions of health [33]. Smokers tend to see the risks of smoking as being lower which may lead to the denial of health problems and to delays in seeking care or preventive services [43,46].

The increasing prevalence of overweight and obesity has become one of the most serious health risks in the modern world [8,14,15]. Overweight and obesity are mainly related to cardiovascular diseases such as coronary heart disease, and type 2 diabetes but also to orthopaedic problems and cancers [6,14,47]. Previous studies have demonstrated a strong association between overweight and obesity and the use of primary and specialized care, hospitalizations and the use of emergency care [6,48], thus imposing large and increasing health care costs on society [5,49,50]. In our study, the strongest associations with health care utilization were found for weight problems. Differently from alcohol consumption and smoking, obesity and/or overweight showed a positive and statistically significant association with all types of health care services, though some gender-based exceptions were observed. The stronger association between obesity and the use of health care services among women (except for specialist care) might be explained by the fact that obesity has a genuinely greater impact on women’s health [51]. On the other hand, as the weight categories are defined by using the same cut-off points for both genders, the greater muscle content of men in the same weight categories may be another reason why overweight and obesity were not as strongly related to health care use among men. Obesity has been related to lower levels of taking Pap tests among women [52], which may partly explain the more equal gender pattern of using specialist care in our study. Although only a relatively small proportion of respondents were underweight, this condition was nevertheless strongly related to ambulance calls among women. Underweight may be a risk factor for health in its own right [47], however, weight loss may also happen as a result of disease. Compared to alcohol and tobacco consumption, obesity may be more related to health conditions that are causing subjective discomfort, thus resulting in more visits to general practitioners.

## Conclusions

Smoking and weight problems impose a significant burden for health care utilization independently from other risk factors. This study points to the necessity of evidence based interventions to reduce the prevalence of unhealthy behaviours and related health problems. For example, the implementation of smoke-free legislation in England has been associated with a reduction in the number of myocardial infarction emergency admissions [53]. Taxation and access control have also proved to be effective tools in reducing tobacco and alcohol consumption among the population [38,54], and other fiscal policies might be similarly effective in terms of weight control [55]. Promoting regular physical exercise and a healthy diet with a focus on work or school settings could be other intervention strategies to reduce the obesity epidemic in the general population [15]. The alcohol-related burden on health care services needs further clarification, possibly by using longitudinal study designs.

## Competing interests

The authors declare that they have no competing interest.

## Authors’ contributions

KV and ML conceived and designed the study. KV conducted the data analyses and drafted the manuscript. ML discussed the core ideas and contributed intellectually to all versions of the manuscript. RAK commented on the earlier drafts. All authors read and approved the final manuscript.

## Pre-publication history

The pre-publication history for this paper can be accessed here:

http://www.biomedcentral.com/1471-2458/13/772/prepub
